# Supplementary material for: Non-equilibrium properties of an active nanoparticle in a harmonic potential
Source: Nat Commun. 2021 Mar 26;12:1902. doi: 10.1038/s41467-021-22187-z (PMC7998004; doi:10.1038/s41467-021-22187-z)
Supplement: Supplementary file 1 — Supplementary Information [file 41467_2021_22187_MOESM1_ESM.pdf]

Non-equilibrium Properties of an Active Nanoparticle in a Harmonic Potential (Supplementary Information)

Falko Schmidt,[1] Hana Šípovà-Jungová,[2] Mikael Käll,[2] Alois Würger,[3] and Giovanni Volpe[1,*]

[1] *Department of Physics, University of Gothenburg, SE-41296 Gothenburg, Sweden*
[2] *Department of Physics, Chalmers University of Technology, SE-41296 Gothenburg, Sweden*
[3] *Laboratoire Ondes et Matière d'Aquitaine, Université de Bordeaux & CNRS, F-33405 Talence, France*

(Dated: February 9, 2021)

Here we provide supplementary information on experimental aspects and on the self-propulsion mechanism of our near-spherical nanoparticles.

* giovanni.volpe@physics.gu.se

SUPPLEMENTARY FIGURES

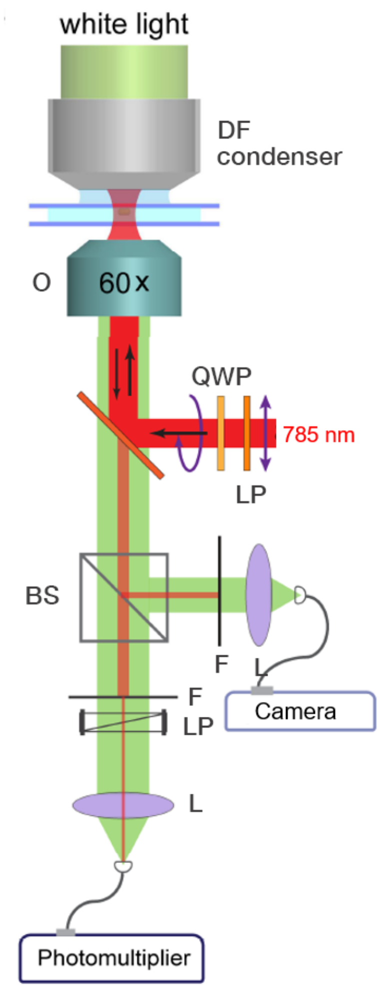

Supplementary Figure 1. Schematic of the experimental setup. A laser beam ($\lambda = 785\,\mathrm{nm}$) passes through a linear polarizer (LP) and a quarter wave plate (QWP) (which permits us to control its polarization from linearly polarized light to right or left-handed circular polarization), before being reflected onto a 60× high-NA objective (O), through which the laser is being focused from below onto the sample chamber. From above, white light illuminates the sample through a dark field (DF) condenser. Only the scattered light of the particle passes and is then split into two paths via a beam splitter (BS). In reflection, the particle's translational motion is recorded using the scattered light focused by a lens (L) into an optical fibre connected to a CMOS camera (the laser light is filtered using by F). In transmission, the particle's spinning motion is recorded using the scattered light passing through a linear polarizer (LP) and collected through a lens (L) by a photomultiplier.

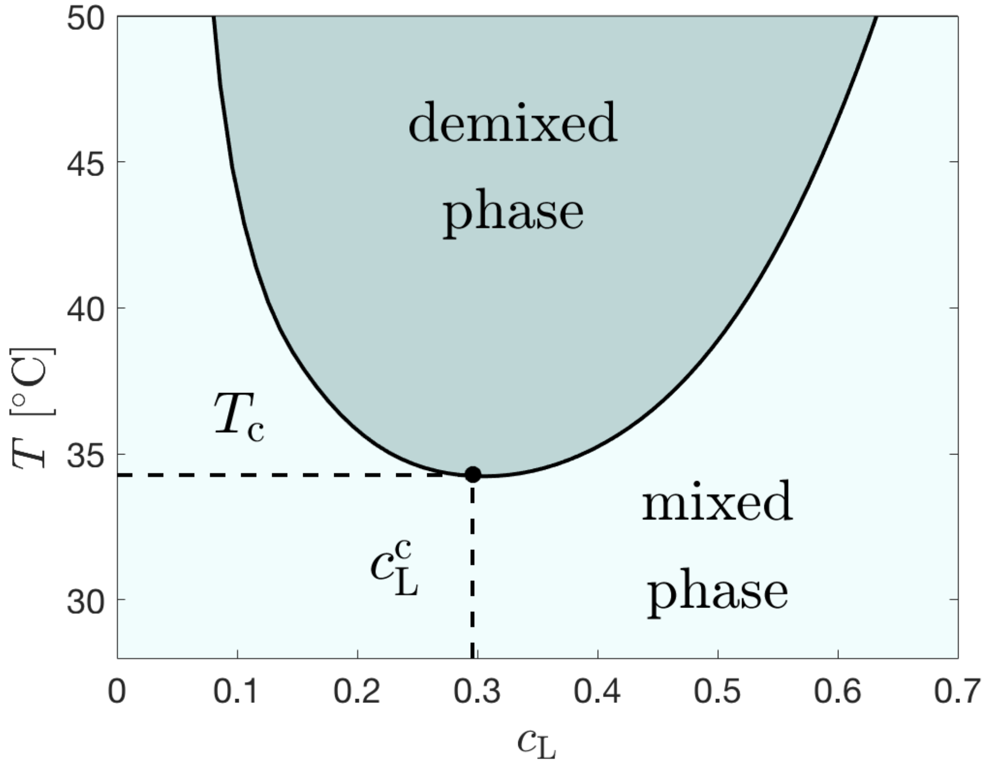

Supplementary Figure 2. Phase diagram of the water–2,6-lutidine mixture. The water–2,6-lutidine mixture is characterized by its spinoidal line (solid line) separating mixed phase (light grey) from demixed phase (dark grey),. The mixture is prepared at the critical lutidine mass fraction $c_\mathrm{c} = 0.286$ and at a temperature $T_0 = 3$°C far away from the critical temperature $T_\mathrm{c} \approx 34$°C. The data for this phase diagram are obtained from Ref. [10].

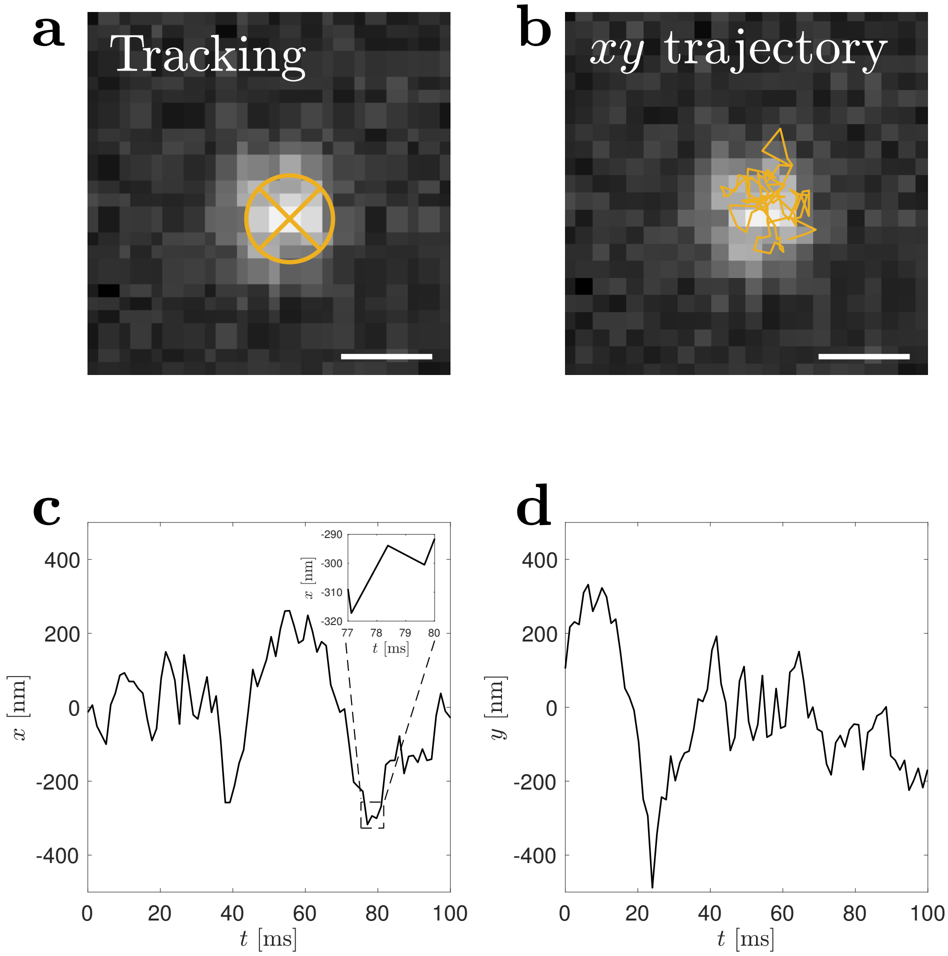

Supplementary Figure 3. Performance of the tracking algorithm. a Example image of a nanosphere with $a = 75\,\mathrm{nm}$ and at fixed laser power $P = 4.36\,\mathrm{mW}$. Using the radial-symmetry particle-tracking algorithm [9], the particle's center is precisely determined and b the xy trajectory reconstructed (trajectory length $t = 100\,\mathrm{ms}$). c, d x- and y-trajectory for $t = 100\,\mathrm{ms}$, respectively. Inset: step sizes down to $5\,\mathrm{nm}$. Image size $22{\times}22\,\mathrm{px}$. The scale bar represents $600\,\mathrm{nm}$. See also Supplementary Video 1.

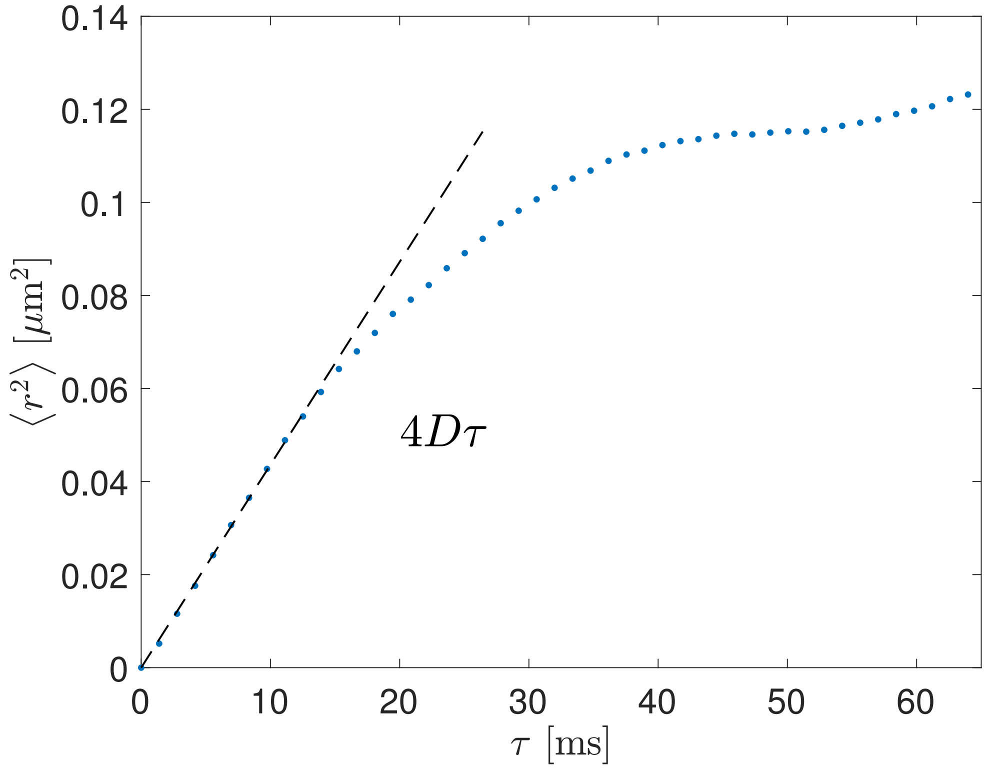

Supplementary Figure 4. Mean-square-displacement of radial position. Mean-square-displacement $\langle r^2 \rangle$ over lag time τ of an exemplary radial trajectory r of a nano sphere with $a = 75\,\mathrm{nm}$ and at fixed laser power $P = 4.36\,\mathrm{mW}$. The dashed line is the linear fit $4D\tau$ at short time scales with $D = 1.09\,\mu\mathrm{m}^2\mathrm{s}^{-1}$.

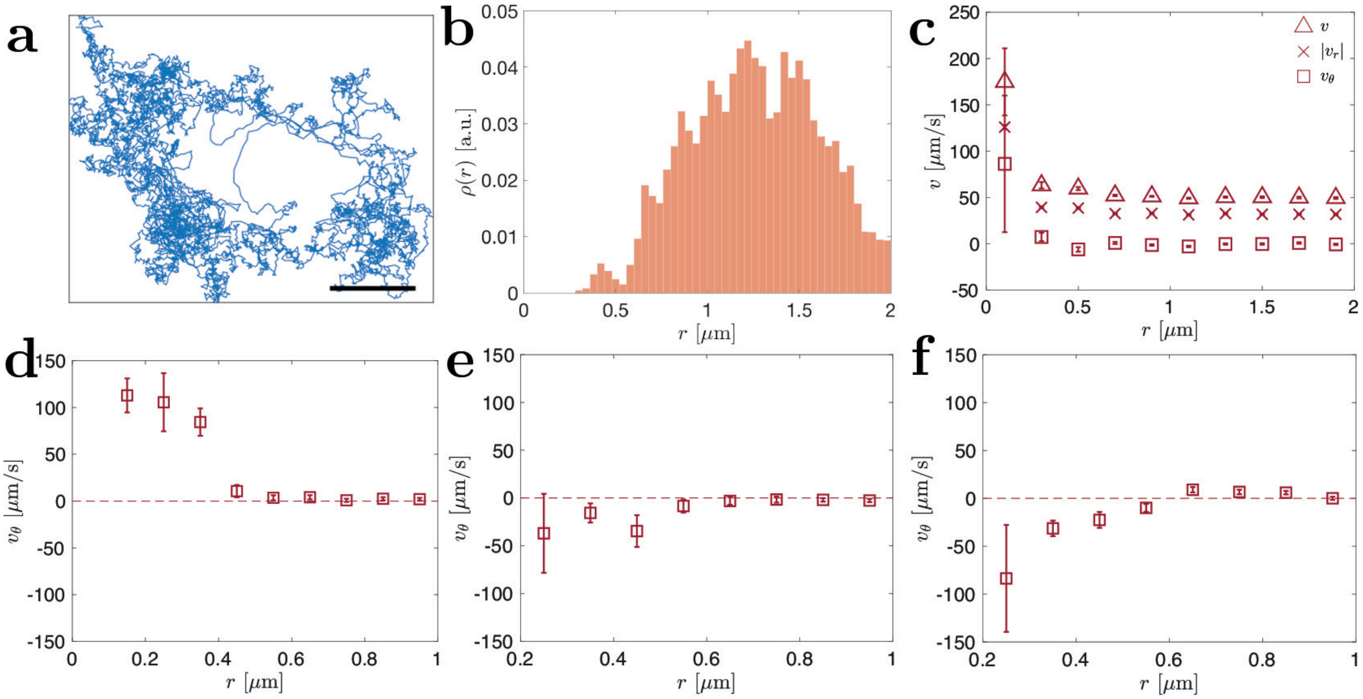

Supplementary Figure 5. Dependence of particle behaviour on shape asymmetry. A nanorod with aspect ratio 1.5 (length $l = 180\,\text{nm}$, width $w = 120\,\text{nm}$) in a critical binary mixture and at laser power $P = 1.1\,\text{mW}$ shows clear out-of-equilibrium behaviour, where the trajectory in a shows the particle moving around the center of the trapping beam, which is reflected in b where the probability distribution is shifted far away from the center. The velocity distributions in c indicate the fast transition of the particle to the beam's periphery where it moves at constant velocities. d-f show fast angular velocities under circular polarization, d $v_\theta > 0$ for left-handed circular polarization, and f $v_\theta < 0$ for right-handed circular polarization, compared to e where $v_\theta \approx 0$ for linear polarization. The scale bar represents $1\,\mu$m. The error is the standard error of the mean.

SUPPLEMENTARY NOTES

Supplementary Note 1: Self-propulsion mechanisms

For laser-heated gold nanoparticles in near-critical water-lutidine we have identified two mechanisms for self-generated motion: thermophoresis is expected to be dominant for temperatures below the lower critical solution point, and diffusiophoresis for temperatures above the lower critical solute point due to varying composition of water-lutidine [1–3]. Other possible mechanisms such as a non-spherical particle shape, non-uniform intensity of the laser beam over the particle surface, and spontaneous symmetry breaking due to a large molecular Péclet number are discussed in detail below.

In summary, we find that a comparison with our experimental observations provides strong arguments for the non-sphericity mechanism, as our estimate for the propulsion velocity u is of the right order and magnitude, and accounts for the rapid motion of the particle in the center of the trap, inward and outwards as well as in azimuthal direction.

Self-thermophoresis of near-spherical particles

We start from the expression for the velocity of a particle in a temperature gradient,

$$u = \frac{1}{\eta}\int_0^\infty dz z h(z) \frac{\langle \nabla_\parallel T \rangle}{T} \tag{1}$$

where $\langle ... \rangle$ is the surface mean, h the excess enthalpy density of the liquid phase due to interactions with the particle, and $\nabla_{\|} T$ the gradient along the surface, which is due to laser heating.

We consider an axisymmetric particle whose surface is parameterized by the cosine $c = \cos\theta$ of the polar angle θ:

$$R(c) = a(1+\chi), \quad \chi = \sum_{n\geq 2} \alpha_n P_n(c), \tag{2}$$

where P_n are Legendre polynomials. The origin is chosen such that $\alpha_1 = 0$.

The temperature field in the liquid satisfies Laplace's equation, $\nabla^2 T = 0$ and thus can be written as a series

$$T = T_0 + \Delta T \frac{a}{r} \left(1 + \sum_{n\geq 1} t_n P_n(c) \frac{a^n}{r^n} \right), \tag{3}$$

where ΔT is the excess temperature with respect to the bulk liquid. For a spherical particle, the coefficients t_n are zero. Here, we determine the temperature field of a non-spherical particle with isothermal surface because the high thermal conductivity of gold imposes a constant surface temperature

$$T(R(c), c) = T_0 + \Delta T. \tag{4}$$

The coefficients t_n are readily obtained by expanding the factors R^{-n} in powers of χ and then projecting the condition (Supplementary Eqn. 4) on P_n. To leading order in α_n, the first coefficient is quadratic in the surface perturbation,

$$t_1 = \sum_{n=2}^{\infty} \frac{3n+2}{2n+3} \alpha_n \alpha_{n+1} \equiv \alpha^2, \tag{5}$$

whereas the remainder of the series contains linear terms,

$$t_n = \alpha_n + O(\alpha^2) \quad (n \geq 2). \tag{6}$$

The temperature at the particle surface is constant as imposed by the isothermal condition (Supplementary Eqn. 4), yet at a distance z the temperature is modulated according to the Fourier series (Supplementary Eqn. 3). Thus $\nabla_{\|} T$ vanishes at $z = 0$ but is finite for $z > 0$. In linear order in χ, only the first Fourier coefficient t_1 contributes to the surface average in (Supplementary Eqn. 1),

$$\nabla_{\|} T = \mathbf{e}_\theta \frac{2 z t_1 \Delta T}{(a+z)^2} \sin\theta + ... \tag{7}$$

Because of the z-dependence of the gradient, the analysis differs from the usual description of self-propulsion.

The excess enthalpy is expressed through the van der Waals energy density,

$$h(z) = -\frac{H}{z^3}, \tag{8}$$

with the Hamaker constants H and where a lower cut-off distance is provided by the molecular size ϵ. Projecting the temperature gradient on the particle axis, averaging over the surface, and performing the integral over z, we find the particle velocity

$$u = -\frac{4\alpha^2 H}{3\eta a^2} \frac{\Delta T}{T} \ln \frac{a}{\epsilon}. \tag{9}$$

There is another contribution which results from the coupling of the higher temperature multipoles t_n to the shape-induced perturbation of the hydrodynamic velocity field. Following Happel and Brenner [4], the rather intricate boundary conditions at a non-spherical particle are implemented in terms of the stream function

$$\frac{\psi}{u a^2} = \left(r_a^2 + \frac{B_2}{r_a} \right) \mathscr{I}_2 + \sum_{n\geq 3} \left(\frac{B_n}{r_a^{n-1}} + \frac{D_n}{r_a^{n-3}} \right) \mathscr{I}_n, \tag{10}$$

with a velocity scale u, the reduced coordinate $r_a = r/a$ and the Gegenbauer functions $\mathscr{I}_n(\cos\theta)$. The resulting velocity corrections are proportional to $\alpha_n \alpha_{n+1}$, like t_1, and of similar order of magnitude.

We start from the expression for the particle velocity derived in previous work on carbon-capped Janus particles [1],

$$u = -\frac{k_{\rm B}T}{\bar{v}\eta}\int_0^\infty dzz\left\langle\frac{e^{-\psi_{\rm w}}-e^{-\psi_{\rm l}}}{\phi e^{-\psi_{\rm w}}+(1-\phi)e^{-\psi_{\rm l}}}\nabla_{\|}\phi\right\rangle, \tag{11}$$

where $k_BT\psi_{w,l}$ are excess interaction potentials of water and lutidine, $\bar{v}^{-1}$ is the mean inverse molecule, and z the distance from the surface. Angular brackets indicate the surface average.

Far from the hot particle, the mixture is at the critical value for the water content $\phi_{\rm c}$. Above the critical temperature $T_{\rm c}$, the local equilibrium composition $\phi(T(\mathbf{r}))$ is given by the spinodal line of the phase diagram and thus depends on the local temperature $T(\mathbf{r})$, which in turn is solution of the heat diffusion equation around the hot particle. Hydrophilic surfaces attract water, resulting within the critical droplet in a water content larger than the critical value, $\phi > \phi_{\rm c}$.

Active motion requires a perturbation with respect to ϕ. In the above expression (Supplementary Eqn. 11), the interaction potentials $\psi_{{\rm w}/l}$ of the particle with nearby water and lutidine molecules result in an out-of-equilibrium composition within an interaction layer. For hydrophilic surfaces, $\psi_{\rm w}$ is more negative than $\psi_{\rm l}$, resulting in an enhancement of the water content beyond ϕ. This non-uniform excess water content along the surface, provides the thermodynamic force which drives the particle. The relation to the temperature gradient is given by

$$\nabla\phi = \phi_{\rm T}\frac{\nabla T}{T}. \tag{12}$$

The remaining analysis is analogous to the case of thermophoresis treated above. The rather intricate phase behavior of the critical mixture is condensed in the parameter $\phi_{\rm T}$, [1] which will be taken as a constant in the following.

The interaction potentials of water and lutidine with the particle surface is described by dispersion forces

$$\psi_{{\rm w}/l} = -\frac{H_{{\rm w}/l}}{k_BT}\frac{\bar{v}}{z^3}, \tag{13}$$

with effective Hamaker constants $H_{{\rm w}/l}$. Linearizing the exponentials in (11), and integrating as above, we find

$$u = \alpha^2\frac{H_{\rm w}-H_{\rm l}}{\eta a^2}\frac{\Delta T_{\rm c}}{T}\phi_{\rm T}\ln\frac{a}{\epsilon} \tag{14}$$

with $\Delta T_{\rm c} = T_{\rm S} - T_{\rm c}$. (The particle is active only at temperatures above T_c.) The velocity is similar to that for thermophoresis, with an additional factor $\phi_{\rm T}$. Previous experiments show that self-propulsion of Janus particles in water-lutidine above T_c is much faster than in water [2, 5], implying that the parameter ϕ_T is much larger than unity. This is confirmed by a mean-field treatment of the spinodals above T_c [1] and numerical simulations [3]. That is why we neglect the thermophoretic contribution. With the notable exception of electric double-layer forces [6], the thermal and chemical surface forces driving active particles cannot be calculated quantitatively; the above numbers for the parameters $H_w - H_l \sim k_BT$, $\alpha^2 \sim 0.06$ and $\phi_T \sim 5$ are reasonable estimates, in line with previous experiments [2, 5].

The particle's excess temperature with respect to the critical point of water-lutidine arises from the absorption of laser light, and reads explicitly

$$T(r) - T_c = \frac{a^2\beta}{3\kappa}\left(Pg(r) - P_{\rm c}\right), \tag{15}$$

with the beam profile $g = e^{-\frac{r^2}{2\sigma^2}}$, the absorption coefficient β, the heat conductivity of the liquid κ, the laser power P, and the critical value P_c where T_c is attained. For further convenience we rewrite the self-propulsion velocity as

$$u(r) = \begin{cases} C(Pg - P_{\rm c}) & \text{for } r < r_{\rm c}, \\ 0 & \text{for } r > r_{\rm c}, \end{cases} \tag{16}$$

where the various parameters are subsumed in

$$C = \alpha^2\frac{H_{\rm w}-H_{\rm l}}{\eta}\phi_{\rm T}\frac{\beta}{3\kappa T}\ln\frac{a}{\epsilon}. \tag{17}$$

For a numerical estimate we put $H_\mathrm{w}-H_\mathrm{l}\approx k_\mathrm{B}T$, $a=75$ nm, $\eta\approx 2$ mPa.s, $\ln(a/d_0)\sim 5$, $\Delta T\sim 50$ K. With the Fourier component $\alpha_n=0.1$, we find that each single mode n contributes the velocity

$$u_n \sim 40\,\mu\mathrm{m/s}. \tag{18}$$

Assuming several modes to be relevant, this estimate accounts for the highest velocities measured. Like for thermophoresis, there is an additional contribution from hydrodynamic corrections, which merely enhance the numerical prefactor, but do not modify the overall features and functional dependencies of the above velocity.

Phoresis in a non-uniform laser beam

Here we calculate the self-propulsion velocity of a spherical particle due to the intensity profile $P(\mathbf{r})$ of the laser beam. The heat absorption rate in the particle is given by $q=\beta P(\mathbf{r}+\hat{\mathbf{r}})$, with the position $\mathbf{r}$ of the particle center and $\hat{\mathbf{r}}$ the distance with respect to the center. Linearizing in terms of $\hat{\mathbf{r}}$ we find

$$q=\beta\left[P(\mathbf{r})+\hat{\mathbf{r}}\cdot\nabla P(\mathbf{r})+...\right], \tag{19}$$

where higher-order terms carry additional powers of the ratio of the particle radius and the beam waist, a/σ. The stationary temperature field T is determined by Fourier's law

$$\kappa\nabla^2 T+q=0, \tag{20}$$

with the thermal conductivity κ and where $q=0$ outside the particle. The boundary conditions at the interface require continuity for both the temperature T and the heat current $-\kappa\mathbf{n}\cdot\nabla T$ in normal direction. We readily obtain the excess temperature inside the particle,

$$T_\mathrm{p}-T_0=\frac{\beta P}{3\kappa_\mathrm{s}}\left(1+\epsilon\frac{a^2-\hat{r}^2}{2a^2}\right)+\frac{\beta\hat{\mathbf{r}}\cdot\nabla P}{10\kappa_\mathrm{p}}\left(\frac{3+2\epsilon}{1+2\epsilon}-\frac{\hat{r}^2}{a^2}\right), \tag{21}$$

and in the outside liquid

$$T_s-T_0=\frac{\beta P}{3\kappa_\mathrm{s}}\frac{a^2}{\hat{r}^2}+\frac{\beta\hat{\mathbf{r}}\cdot\nabla P}{5\kappa_\mathrm{p}}\frac{1}{1+2\epsilon}\frac{a^3}{\hat{r}^3}, \tag{22}$$

where we use the thermal conductivity contrast between solvent κ_s and particle κ_p, $\epsilon=\kappa_\mathrm{s}/\kappa_\mathrm{p}$. The conductivity of gold being about thousand times larger than that of water and oil, we put $\epsilon=0$ in the following.

Phoresis is driven by the component of the temperature gradient that is parallel to the particle surface,

$$\nabla_\parallel T=\frac{\beta(1-\mathbf{nn})\cdot\nabla P(\mathbf{r})}{5\kappa_\mathrm{p}}. \tag{23}$$

Proceeding as above, we readily obtain the diffusiophoretic self-propulsion velocity

$$u_\mathrm{grad}=\frac{\kappa_\mathrm{s}}{\kappa_\mathrm{p}}\frac{H_\mathrm{w}-H_\mathrm{l}}{\eta a}\frac{\Delta T}{T}\phi_\mathrm{T}\frac{r}{\sigma^2}e^{-r^2/2\sigma^2}. \tag{24}$$

With the above numbers and the heat conductivity ratio of water and gold, $\kappa_\mathrm{s}/\kappa_\mathrm{p}\approx 10^{-3}$, we find a velocity much smaller than one micron per second.

Moreover, u_grad vanishes at center of the beam, where the experiments show the highest velocities. Finally, u_grad is always aligned on the radial direction, whereas the observations show equally fast motion in polar direction. We conclude that the contribution proportional to the intensity gradient does not provide a major contribution to the active motion of our particles.

Spontaneous symmetry breaking

Michelin et al. [7] have shown that active motion of spherical particles may occur due to spontaneous symmetry breaking of the molecular composition of the surrounding solution.. This nonlinear mechanism sets as soon as the molecular Péclet number $\mathrm{Pe_m}=au/D_\mathrm{m}$ exceeds a critical value of 4; in other words, the lowest velocity expected is of the order of $u_\mathrm{min}=4D_\mathrm{m}/a$, which is larger than 1 mm/s. Yet our experiments show active motion with $u_0\sim 25\mu$m/s, thus ruling out spontaneous symmetry breaking.

More importantly, the nonlinear driving mechanism works only if the excess molecular species is repelled from the particle surface. (In the language of Ref. [7], activity and mobility need to carry the same sign.) This is, however, never the case for particles in near-critical water-lutidine, since the excess molecular species is the one being attracted by the surface. As a consequence, non-linear effects at high Péclet number are not expected to result in self-propulsion.

SUPPLEMENTARY NOTE 2: EXPERIMENTAL DETAILS

Here, we provide more experimental details on the particle tracking and the influence of shape asymmetry on the particle's motion.

Particle Tracking

We have imaged the nanoparticle in a commercial inverted microscope setup using a dark field condenser (see Supplementary Figure 1). Due to localized surface plasmon resonances (LSPR) on the particle's metallic surface, incident white light is strongly scattered leading to an increased scattering cross section [8]. The resulting image of the particle is visible as bright white spot on the camera. We tracked the particle's motion with sub-pixel resolution using radial symmetry calculations [9]. Supplementary Figure 3 provides an example image of the particle that appears about 6 bigger than its actual size and indicates the center of the tracked particle (see also Supplementary Video 1), as well as reconstructed xy trajectories and the individual trajectories along x and y direction, respectively.

Dependence on particle shape

We have further investigated the dependence of the particle's behaviour on its shape and employed nanorods with aspect ratio of 1.5. The larger nanorods (length $l = 180\,\mathrm{nm}$, width $w = 120\,\mathrm{nm}$) already absorb enough light at $P = 1.1\,\mathrm{mW}$ to induce strong demixing that pushes the particle out of the center of the trapping beam. In Supplementary Figures 5a illustrates how the particle moves in the periphery of the beam and only rarely passes through the center. The probability and velocity distributions (Supplementary Figures. 5b,c, respectively) emphasize this behaviour where a clear out-of-equilibrium signature can be found. We have further investigated the dependence of the angular velocity v_θ on circular polarization of the light beam and find similar results as for our nanosphere although at much higher speeds (about 10x larger compared to the nanosphere). This shows that particle shape plays a significant role in the resulting behaviour and where asymmetry can greatly improve rotation speeds of future nanomotors.

SUPPLEMENTARY REFERENCES

[1] Würger, A. Self-diffusiophoresis of Janus particles in near-critical mixtures. *Phys. Rev. Lett.* **115**, 188304 (2015).

[2] Buttinoni, I., Volpe, G., Kümmel, F., Volpe, G. & Bechinger, Clemens Active Brownian motion tunable by light. *J. Phys.: Condens. Matter* **24**, 284129 (2012).

[3] Samin, S & Van Roij, R. Self-propulsion mechanism of active Janus particles in near-critical binary mixtures. *Phys. Rev. Lett.* **115**, 188305 (2015).

[4] J. Happel, H. Brenner. *Low Reynolds number hydrodynamics.* Martinus Nijhoff, Massachusetts, 1963.

[5] Lozano, Celia , Gomez-Solano, Juan Ruben, & Bechinger, Clemens Run-and-tumble-like motion of active colloids in viscoelastic media. *New J. Phys.* **20**, 015008 (2018).

[6] Eslahian, Kyriakos A., Majee, Arghya, Maskos, Michael & Würger, Alois Specific salt effects on thermophoresis of charged colloids. *Soft Matter* **10**, 1931-1936 (2014).

[7] Michelin, S, Lauga, E. & Bartolo, D. Spontaneous autophoretic motion of isotropic particles. *Phys. Fluids* **25**, 061701 (2013).

[8] Liu, Mengmeng, Chao, Jie, Deng, Suhui, Wang, Kun, Li, Kun & Fan, Chunhai Dark-field microscopy in imaging of plasmon resonant nanoparticles. *Colloids Surf. B* **124**, 111–117 (2014).

[9] Parthasarathy, Raghuveer Rapid, accurate particle tracking by calculation of radial symmetry centers. *Nat. Methods* **9**, 724–726 (2012).

[10] Grattoni, C. A., Dawe, R. A., Seah, C. Y. & Gray, J. D. Lower critical solution coexistence curve and physical properties (density, viscosity, surface tension, and interfacial tension) of 2,6-lutidine + water. *J. Chem. Eng. Data* **38**, 516 (1993).
