# Supplementary material for: Non-equilibrium properties of an active nanoparticle in a harmonic potential
Source: Nat Commun. 2021 Mar 26;12:1902. doi: 10.1038/s41467-021-22187-z (PMC7998004; doi:10.1038/s41467-021-22187-z)
Supplement: Supplementary file 3 — Description of Additional Supplementary Files [file 41467_2021_22187_MOESM3_ESM.pdf]

Description of Additional Supplementary Files

File name: Supplementary Movie 1
Description: Tracking of a nanoparticle. Recorded dark field images of a nanoparticle with radius a = 75 nm at fixed laser power P = 4.44 mW). Using a radial symmetry tracking particle's position is determined. Video is played at 0.1 times the original acquisition rate
